# Efficacy and safety of antibody-drug conjugates in EGFR-mutant non-small cell lung cancer after tyrosine kinase inhibitor resistance: a systematic review and meta-analysis

**DOI:** 10.3389/fonc.2026.1832920

**Published:** 2026-07-16

**Authors:** Xiao Ma, Gaofeng Li, Heng Li

**Affiliations:** Department of Thoracic Surgery II, The Third Affiliated Hospital of Kunming Medical University/Yunnan Cancer Hospital/Peking University Cancer Hospital Yunnan Branch, Kunming, China

**Keywords:** antibody-drug conjugates, EGFR mutation, HER3, meta-analysis, non-small cell lung cancer, TROP2, tyrosine kinase inhibitor resistance

## Abstract

**Background:**

Patients with epidermal growth factor receptor (EGFR)-mutant non-small cell lung cancer (NSCLC) inevitably develop resistance to EGFR-tyrosine kinase inhibitors (TKIs). Antibody-drug conjugates (ADCs) have emerged as a promising therapeutic strategy in this setting; however, no meta-analysis has systematically evaluated ADC efficacy and safety specifically in this population.

**Methods:**

We systematically searched PubMed, Embase, the Cochrane Library, and Web of Science for clinical trials evaluating ADCs in patients with EGFR-mutant NSCLC after TKI failure, published up to December 2025. The primary endpoint was objective response rate (ORR). Secondary endpoints included median progression-free survival (mPFS), median overall survival (mOS), and hazard ratios (HR) for PFS and OS in randomized controlled trials (RCTs). Pooled proportions were estimated using the Freeman-Tukey double arcsine transformation with a DerSimonian-Laird random-effects model, and subgroup analyses were stratified by ADC target antigen. A random-effects meta-regression was used to assess whether the median number of prior treatment lines explained heterogeneity.

**Results:**

Nine studies encompassing 1,092 patients were included. The pooled ORR was 44.7% (95% CI 36.7%–52.9%; I² = 84.4%), with substantial heterogeneity driven by between-target differences. Subgroup analysis demonstrated a significantly higher ORR for TROP2-targeting ADCs (50.6%; 95% CI 40.3%–60.9%) than for HER3-targeting ADCs (34.2%; 95% CI 28.8%–39.8%) (P for interaction = 0.006). Among three RCTs, the two trials of sacituzumab tirumotecan (Sac-TMT) versus chemotherapy yielded a pooled HR of 0.40 (95% CI 0.25–0.64) for PFS and 0.57 (95% CI 0.44–0.76; I² = 0%) for OS, both favouring ADC therapy; by contrast, the phase III HERTHENA-Lung02 trial of patritumab deruxtecan (HER3-DXd) improved PFS (HR 0.77; 95% CI 0.63–0.94; P = 0.011), but OS data were immature at the time of the analysis. Across studies, mPFS ranged from 5.5 to 11.1 months, and mature mOS data were available from only three studies (range 11.9–16.2 months).

**Conclusions:**

ADCs demonstrate substantial antitumour activity in EGFR-mutant NSCLC after TKI resistance, but efficacy appears target- and drug-specific rather than a uniform class effect. Among the agents studied, only the TROP2-ADC sacituzumab tirumotecan has shown improvement in both PFS and OS over chemotherapy, whereas the HER3-ADC patritumab deruxtecan improved PFS without an OS benefit. Given high between-study heterogeneity and OS immaturity in most studies, these findings support ADCs—particularly TROP2-directed agents—as a key therapeutic option for this difficult-to-treat population, while underscoring the need for biomarker-guided patient selection.

## 1 Introduction

Non-small cell lung cancer (NSCLC) accounts for approximately 80–85% of all lung cancers and remains a leading cause of cancer-related mortality worldwide, with an estimated 2.20 million new cases and 1.79 million deaths annually (1, 2). Mutations in the epidermal growth factor receptor (EGFR) gene constitute the most prevalent actionable oncogenic driver in NSCLC, with a frequency that varies markedly by ethnicity—approximately 50% in East Asian populations versus 13–15% in Western populations (3, 4). The successive development of EGFR-tyrosine kinase inhibitors (TKIs)—from first-generation agents (gefitinib, erlotinib) to third-generation osimertinib—has transformed the treatment paradigm for advanced EGFR-mutant NSCLC, achieving response rates of 50–80% and substantially prolonging progression-free survival (PFS) compared with platinum-based chemotherapy (5–7).

Despite these advances, acquired resistance to EGFR-TKIs is virtually inevitable. The mechanisms are diverse and include on-target EGFR mutations (e.g., T790M and C797S) (8), activation of bypass signalling pathways such as MET and HER2 amplification—MET amplification driving resistance via ERBB3-dependent signalling (9, 10)—and histological transformation to small cell lung cancer (11–14). After progression on osimertinib and platinum-based chemotherapy, treatment options become severely limited; standard subsequent-line chemotherapy yields a median PFS of only 2.8–3.2 months and a median OS of 9.6–12.3 months (15, 16).

Antibody-drug conjugates (ADCs) represent a class of oncologic therapeutics that combine the targeting specificity of monoclonal antibodies with the cytotoxic potency of chemotherapeutic payloads through engineered linkers (17). By targeting tumour-associated antigens—including HER3, TROP2, c-MET, HER2, and EGFR itself—ADCs can circumvent many EGFR-TKI resistance mechanisms, induce DNA damage independently of EGFR signalling, and elicit bystander effects against heterogeneous resistant clones (18, 19).

In recent years, multiple ADCs have demonstrated encouraging clinical activity in EGFR-mutant NSCLC following TKI failure. The phase III OptiTROP-Lung04 trial established sacituzumab tirumotecan (Sac-TMT) as the first ADC to demonstrate both PFS and OS improvements over chemotherapy in this population (20). The phase II HERTHENA-Lung01 trial showed clinically meaningful activity of patritumab deruxtecan (HER3-DXd) (21), and the first-in-class EGFR×HER3 bispecific ADC BL-B01D1 has exhibited promising preliminary antitumour activity (22). More recently, the phase III HERTHENA-Lung02 trial reported that HER3-DXd significantly improved PFS versus platinum-based chemotherapy in EGFR-mutant NSCLC after a third-generation TKI, with OS data immature at the interim analysis (23); this provides the first randomized comparison for a HER3-ADC in this setting.

Despite this rapidly evolving landscape, no systematic review or meta-analysis has comprehensively assessed the efficacy and safety of ADCs specifically in the EGFR-mutant NSCLC population after TKI resistance. Prior meta-analyses have focused on biomarker-unselected populations (24, 25), individual targets such as HER2 alone (26), safety endpoints exclusively (27), or ADC-versus-chemotherapy comparisons without EGFR-mutant subgroup specificity (28). The present study addresses this gap by performing the first systematic review and meta-analysis encompassing all ADCs evaluated in EGFR-mutant NSCLC following TKI failure across multiple ADC targets.

## 2 Materials and methods

### 2.1 Search strategy and registration

This systematic review and meta-analysis was conducted in accordance with the PRISMA 2020 guidelines (29). A comprehensive literature search was performed across PubMed, Embase, the Cochrane Library, and Web of Science from database inception through December 31, 2025. The search combined MeSH terms and free-text keywords for ADCs (including specific agent names) AND NSCLC AND EGFR/TKI resistance. Conference abstracts from ASCO and ESMO annual meetings (2020–2025) were also reviewed.

### 2.2 Eligibility criteria

Population: adults with histologically or cytologically confirmed EGFR-mutant advanced/metastatic NSCLC progressing after at least one EGFR-TKI. Intervention: any ADC as monotherapy or combined with a TKI. Comparator: not required for single-arm studies; chemotherapy or placebo for RCTs. Outcomes: ORR, PFS, OS, or DCR. Study design: prospective trials (phase I–III). *Post-hoc* EGFR-mutant subgroup analyses were eligible only when independently reported with sufficient detail (sample size, responders, survival). On this basis, EVOKE-01 (sacituzumab govitecan vs docetaxel) was not eligible for quantitative pooling because its EGFR-mutant subgroup was not independently reported with sufficient outcome detail; it is discussed qualitatively (§4.1). Exclusion criteria comprised preclinical studies, case series with fewer than 10 patients, reviews/editorials, and non-English publications.

### 2.3 Study selection and data extraction

Two investigators independently screened records and performed full-text assessment, with disagreements resolved by a third reviewer. Extracted variables included study name, identifier, author/year, phase, ADC and target, EGFR-mutant sample size, responders, ORR, mPFS, mOS, DCR, hazard ratios, prior treatment lines, and key adverse events. The most recent comprehensive data cut was used when populations were reported multiple times.

### 2.4 Risk of bias assessment

Risk of bias was assessed using the Cochrane RoB 2 tool (30) for RCTs and the modified Newcastle-Ottawa Scale (31) for single-arm studies.

### 2.5 Statistical analysis

The primary outcome was pooled ORR as a proportion. The Freeman-Tukey double arcsine transformation (32) was applied to stabilise variance, and pooled estimates were calculated using the DerSimonian-Laird random-effects model (33), with back-transformation using the harmonic mean of sample sizes. Study-specific 95% CIs used the Clopper-Pearson exact method. For RCTs, pooled HRs for PFS and OS were calculated using the generic inverse-variance random-effects method (34). Heterogeneity was evaluated using Cochran’s Q and the I² statistic (35), and subgroup analyses were pre-specified by ADC target antigen, with between-subgroup differences assessed by the Q test for interaction.

To explore sources of heterogeneity, a random-effects meta-regression was performed with the Freeman-Tukey-transformed ORR as the dependent variable and the median number of prior treatment lines per study as a study-level covariate. All analyses were performed in Python 3.12 (SciPy v1.11, NumPy v1.26) and verified with the metafor package (v4.4) in R (v4.3.2). A two-sided P < 0.05 was considered significant.

## 3 Results

### 3.1 Study selection

**Figure 1:**
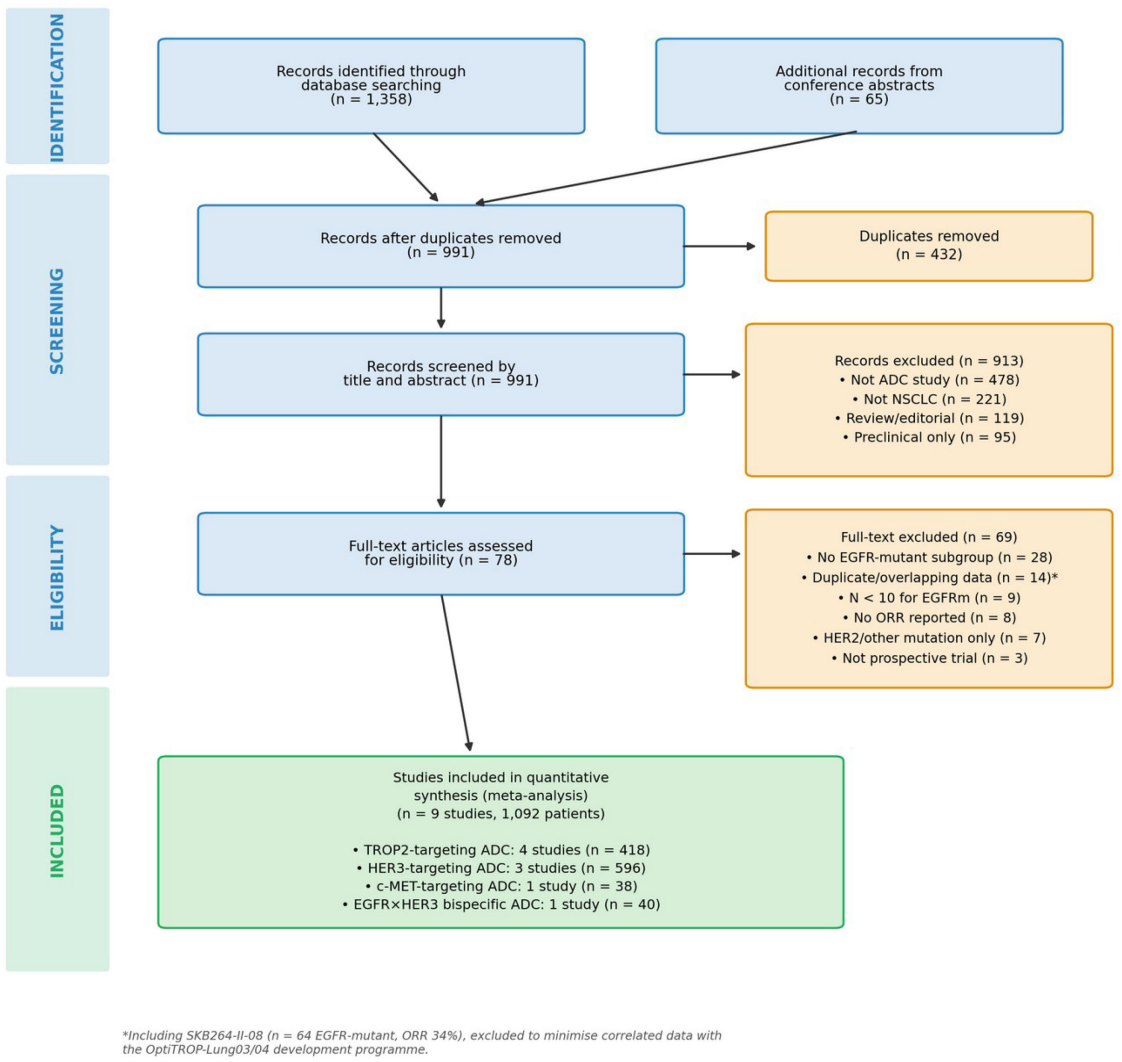
PRISMA 2020 flow diagram of study selection. Nine studies encompassing 1,092 patients with EGFR-mutant NSCLC were included in the final analysis.

The systematic search identified 1,423 records (1,358 from databases and 65 from conference proceedings). After removal of 432 duplicates, 991 records were screened, of which 78 underwent full-text review. Ultimately, nine studies met the inclusion criteria and were included in the final analysis. The PRISMA flow diagram is presented in **Figure 1**.

### 3.2 Study characteristics

The nine included studies comprised a total of 1,092 patients with EGFR-mutant NSCLC who had progressed after EGFR-TKI therapy (**Table 1**). Studies were published between 2023 and 2025. The ADC targets included TROP2 (4 studies, n = 418), HER3 (3 studies, n = 596), c-MET (1 study, n = 38), and EGFR×HER3 bispecific (1 study, n = 40). Six studies employed single-arm designs (phase I–II), and three were randomized controlled trials: two phase II/III trials comparing sacituzumab tirumotecan with chemotherapy and the phase III HERTHENA-Lung02 trial comparing patritumab deruxtecan with platinum-based chemotherapy.

**Table 1:** Characteristics of included studies (updated to nine studies; the U31402-A-U102 row reports the prior-3G-TKI-plus-PBC subgroup, n = 78, as a table footnote).

Study (trial)	ADC	Target	Phase	EGFRm N	ORR %	mPFS (mo)	mOS (mo)	Prior lines (median)	Design
U31402-A-U102 (36)	HER3-DXd	HER3	I	78	41.0	6.4	16.2	4	Single-arm
HERTHENA-Lung01 (21)	HER3-DXd	HER3	II	225	29.8	5.5	11.9	3	Single-arm
HERTHENA-Lung02 (23)	HER3-DXd	HER3	III	293	35.2	5.8	NR (immature)	1	RCT vs PBC
KL264–01 EGFRm (37)	Sac-TMT	TROP2	I/II	22	54.5	11.1	NR	2	Single-arm
OptiTROP-Lung03 (38, BMJ)	Sac-TMT	TROP2	II	91	45.1	6.9	NR	2	RCT vs docetaxel
OptiTROP-Lung04 (20, NEJM)	Sac-TMT	TROP2	III	188	60.6	8.3	NR	1	RCT vs chemo
Dato-DXd EGFRm pooled (39)	Dato-DXd	TROP2	II/III	117	42.7	5.8	15.6	3	Pooled
Teliso-V + Osimertinib (40)	Teliso-V	c-MET	Ib	38	50.0	7.4	NR	2	Single-arm (combo)
BL-B01D1 EGFRm (22)	BL-B01D1	EGFR×HER3	I	40	52.5	5.7	NR	3	Single-arm

ADC, antibody-drug conjugate; mOS, median overall survival; mPFS, median progression-free survival; NR, not reached; ORR, objective response rate; PBC, platinum-based chemotherapy; Sac-TMT, sacituzumab tirumotecan. The HERTHENA-Lung02 row (highlighted) is new in this revision; its OS was immature at the protocol-specified interim analysis. The U31402-A-U102 ORR (41.0%, 32/78) reflects the prior-3G-TKI-plus-platinum subgroup.

Among the TROP2-targeting ADCs, three studies evaluated Sac-TMT (20, 37, 38) and one reported a pooled analysis of datopotamab deruxtecan (Dato-DXd) (39). All three HER3-targeting studies evaluated patritumab deruxtecan (HER3-DXd) (21, 23, 36). The c-MET study assessed telisotuzumab vedotin with osimertinib (40), and the bispecific study evaluated BL-B01D1 (22). The median number of prior treatment lines ranged from 1 to 4 across studies.

### 3.3 Primary outcome: pooled objective response rate

**Figure 2:**
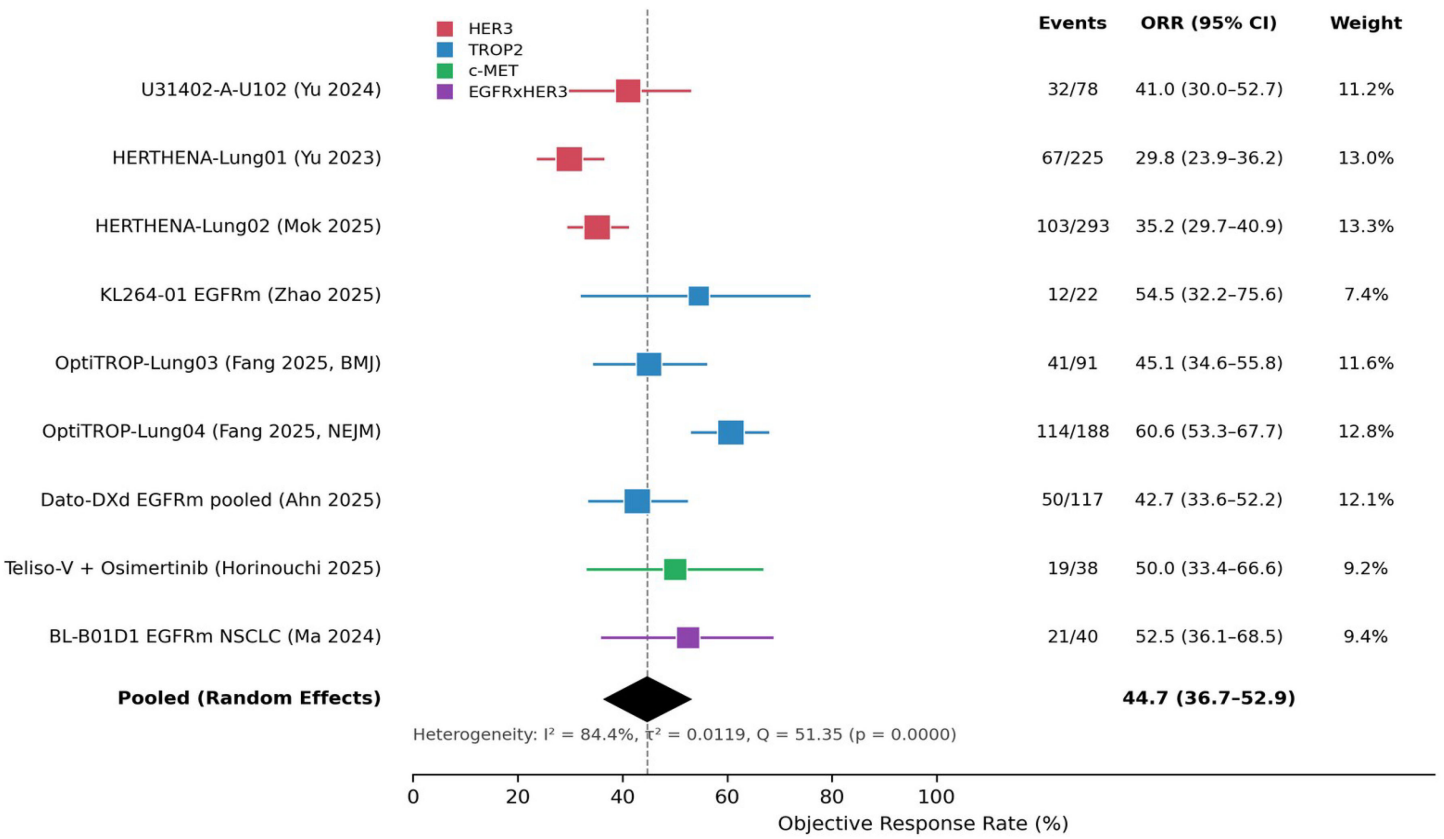
Forest plot of pooled ORR across the nine included studies. Pooled ORR = 44.7% (95% CI 36.7%–52.9%; I² = 84.4%).

The pooled ORR across all nine studies was 44.7% (95% CI 36.7%–52.9%), based on the random-effects model (**Figure 2**). Substantial heterogeneity was observed (I² = 84.4%, Q = 51.35, P < 0.0001), which was anticipated given differences in ADC targets, treatment lines, and study designs. This heterogeneity was primarily driven by between-target differences, as confirmed by the significant subgroup interaction test (P = 0.006). Individual study ORRs ranged from 29.8% (HERTHENA-Lung01, HER3-DXd) to 60.6% (OptiTROP-Lung04, Sac-TMT). The two largest studies—HERTHENA-Lung02 (n = 293) and HERTHENA-Lung01 (n = 225)—contributed the greatest weight to the pooled estimate.

### 3.4 Subgroup analysis by ADC target

Subgroup analysis revealed significant differences in ORR according to ADC target (P for interaction = 0.006).

**Figure 3:**
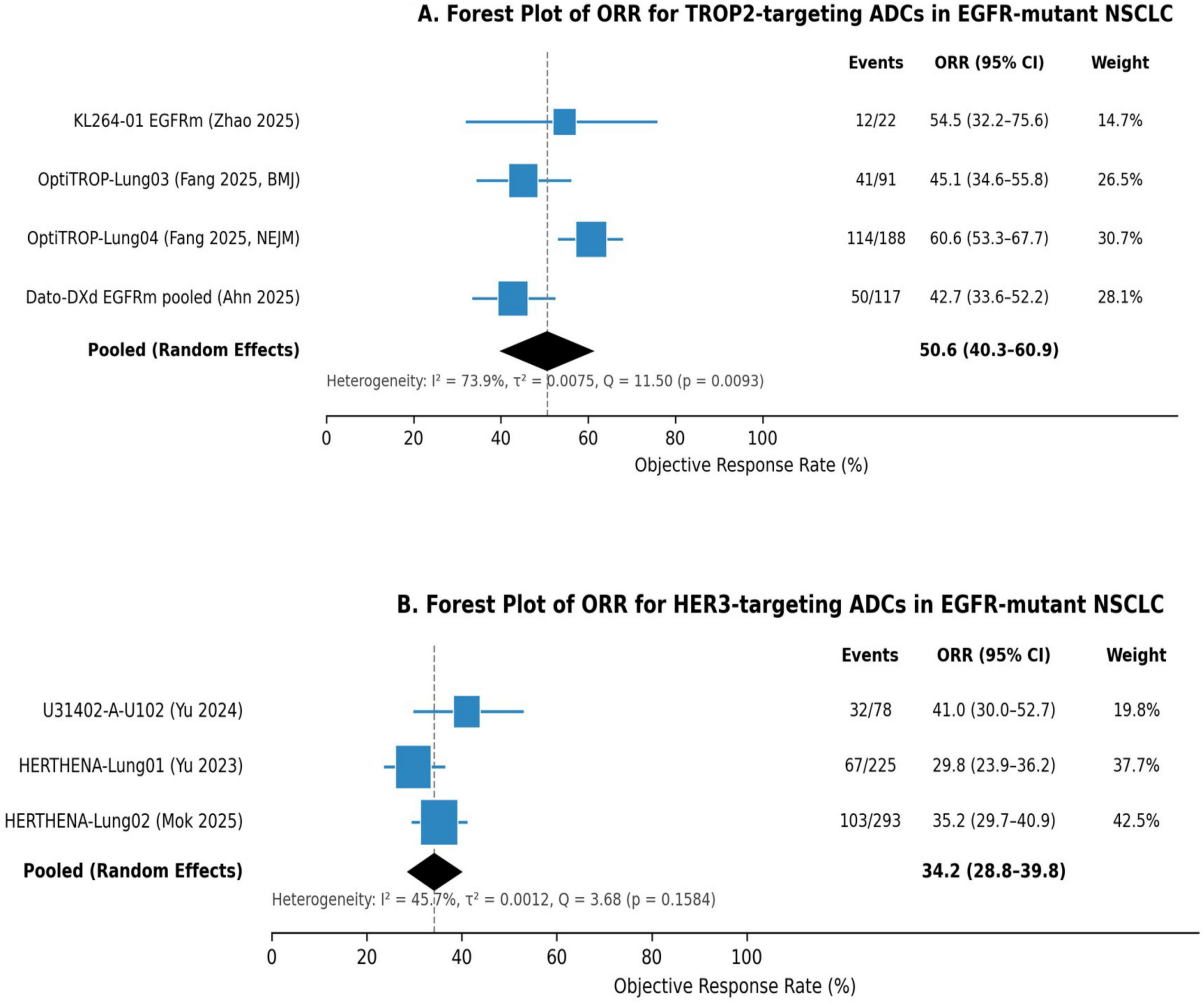
**(A)** Forest plot of pooled ORR for the TROP2-targeting ADC subgroup (4 studies, n = 418). Pooled ORR = 50.6% (95% CI 40.3%–60.9%; I² = 73.8%). **(B)** Forest plot of pooled ORR for the HER3-targeting ADC subgroup (3 studies, n = 596). Pooled ORR = 34.2% (95% CI 28.8%–39.8%; I² = 45.7%).

TROP2-targeting ADCs (4 studies, n = 418): pooled ORR = 50.6% (95% CI 40.3%–60.9%), I² = 73.8%. The highest ORR was observed with Sac-TMT in OptiTROP-Lung04 (60.6%), followed by KL264–01 EGFRm (54.5%, 12/22) and OptiTROP-Lung03 (45.1%). Dato-DXd showed an ORR of 43% (50/117). The TROP2-subgroup forest plot is shown in **Figure 3A**.

HER3-targeting ADCs (3 studies, n = 596): pooled ORR = 34.2% (95% CI 28.8%–39.8%), I² = 45.7%. The phase I U31402-A-U102 study reported 41.0%, the phase III HERTHENA-Lung02 trial 35.2% (103/293), and the larger phase II HERTHENA-Lung01 trial 29.8% in a more heavily pretreated population. The addition of HERTHENA-Lung02 reduced subgroup heterogeneity (from I² = 69.2% to 45.7%) and tightened the pooled estimate. A dedicated HER3-subgroup forest plot is shown in **Figure 3B**.

c-MET-targeting ADC (1 study, n = 38): telisotuzumab vedotin combined with osimertinib achieved an ORR of 50.0%. As the only ADC-plus-TKI study, the individual contribution of each agent cannot be disentangled; a leave-one-out sensitivity analysis excluding this study left the pooled ORR essentially unchanged (44.2%, 95% CI 35.7%–52.9%).

EGFR×HER3 bispecific ADC (1 study, n = 40): BL-B01D1 demonstrated an ORR of 52.5% (21/40), with responses across diverse EGFR-TKI resistance mechanisms.

Meta-regression. In a random-effects meta-regression, the median number of prior treatment lines was not significantly associated with ORR (slope −0.032, SE 0.044; P = 0.47), and residual heterogeneity remained high (Q_res = 46.1). Pretreatment intensity therefore did not statistically account for the observed heterogeneity, which was instead attributable to between-target differences. This analysis was limited by the small number of studies (k = 9).

### 3.5 Survival outcomes

All studies reported mPFS, ranging from 5.5 months (HERTHENA-Lung01) to 11.1 months (KL264–01 EGFRm). Median PFS was 5.8 months for HER3-DXd in HERTHENA-Lung02, 6.4 months for HER3-DXd in U31402-A-U102, 5.8 months for Dato-DXd, 6.9–8.3 months for Sac-TMT in the RCTs, 7.4 months for Teliso-V plus osimertinib, and 5.7 months for BL-B01D1.

Mature mOS data were available from three single-arm/early-phase studies: U31402-A-U102 (16.2 months), the Dato-DXd pooled analysis (15.6 months), and HERTHENA-Lung01 (11.9 months). Randomized OS evidence derives principally from the two Sac-TMT RCTs; in HERTHENA-Lung02, OS data were immature at the protocol-specified interim analysis and no hazard ratio was reported. The reliability of broad survival inferences beyond the randomized Sac-TMT evidence is therefore limited by OS immaturity in the remaining studies.

### 3.6 RCT analysis: ADC vs. chemotherapy

**Figure 4:**
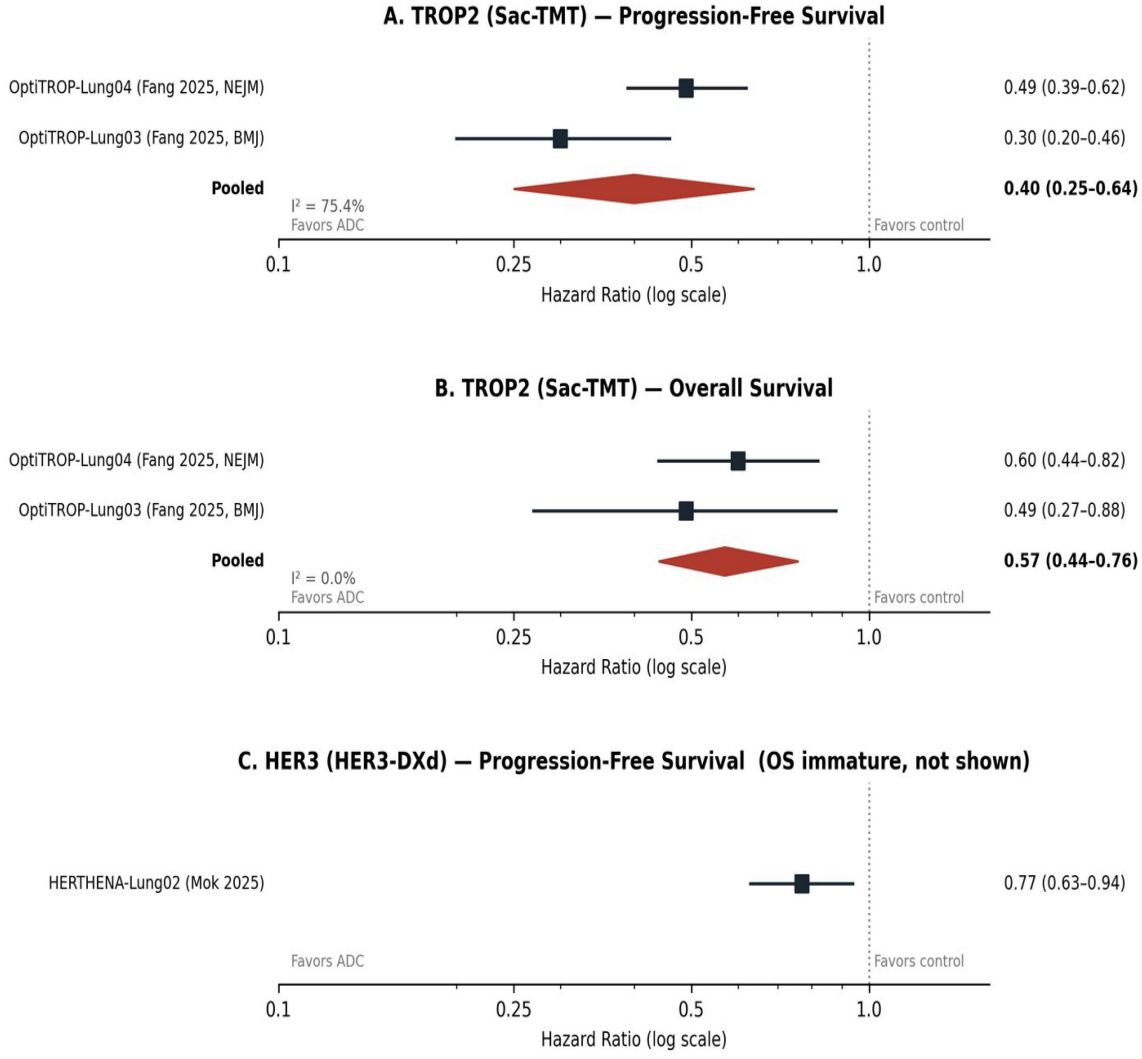
Hazard ratios for PFS and OS from randomized controlled trials, stratified by ADC target. **(A, B)** Pooled TROP2 (Sac-TMT): PFS HR 0.40, OS HR 0.57 (I² = 0%). **(C)** HER3 (HER3-DXd, HERTHENA-Lung02): PFS HR 0.77 (95% CI 0.63–0.94); OS was immature and is not shown.

Three RCTs compared an ADC with chemotherapy in EGFR-mutant NSCLC; because they evaluated different targets, hazard ratios are presented stratified by ADC target (**Table 2**; **Figure 4**).

**Table 2:** Efficacy outcomes of randomized controlled trials comparing ADC versus chemotherapy in EGFR-mutant NSCLC.

Trial	Comparison	ORR (ADC vs ctrl)	mPFS (mo)	PFS HR (95% CI)	OS HR (95% CI)	Phase/source
OptiTROP-Lung04	Sac-TMT vs pemetrexed+platinum	60.6% vs 43.1%	8.3 vs 4.3	0.49 (0.39–0.62)	0.60 (0.44–0.82)	III/NEJM
OptiTROP-Lung03	Sac-TMT vs docetaxel	45.1% vs 17.4%	6.9 vs 2.8	0.30 (0.20–0.46)	0.49 (0.27–0.88)	II/BMJ
**Pooled (Sac-TMT, TROP2)**	**—**	**—**	**—**	**0.40 (0.25–0.64)**	**0.57 (0.44–0.76)**	**I²(OS)=0%**
HERTHENA-Lung02	HER3-DXd vs platinum chemo	35.2% vs 25.3%	5.8 vs 5.4	0.77 (0.63–0.94)	Immature (NR)	III/ASCO 2025

HR, hazard ratio; mPFS, median progression-free survival; NR, not reported; ORR, objective response rate; OS, overall survival; PBC, platinum-based chemotherapy; Sac-TMT, sacituzumab tirumotecan. The HERTHENA-Lung02 row (highlighted) is new in this revision; its OS was immature at the protocol-specified interim analysis. HER3-DXd and Sac-TMT were not pooled together.

Bold values indicate the pooled (meta-analysed) hazard ratios for the Sac-TMT (TROP2) subgroup.

TROP2 (Sac-TMT). OptiTROP-Lung04 (phase III): ORR 60.6% vs 43.1%; PFS 8.3 vs 4.3 months, HR 0.49 (0.39–0.62); OS HR 0.60 (0.44–0.82). OptiTROP-Lung03 (phase II): ORR 45.1% vs 17.4%; PFS 6.9 vs 2.8 months, HR 0.30 (0.20–0.46); OS HR 0.49 (0.27–0.88). The pooled HR was 0.40 (95% CI 0.25–0.64) for PFS and 0.57 (95% CI 0.44–0.76; I² = 0%) for OS, both favouring ADC therapy.

HER3 (HER3-DXd). In the phase III HERTHENA-Lung02 trial, HER3-DXd improved PFS (median 5.8 vs 5.4 months; HR 0.77, 95% CI 0.63–0.94; P = 0.011) and ORR (35.2% vs 25.3%) versus platinum-based chemotherapy; OS data were immature at the protocol-specified interim analysis and no OS hazard ratio was reported. HER3-DXd and Sac-TMT RCT results were not pooled together, given the divergent targets and the absence of mature HER3-DXd OS data.

### 3.7 Sensitivity analysis

**Figure 5:**
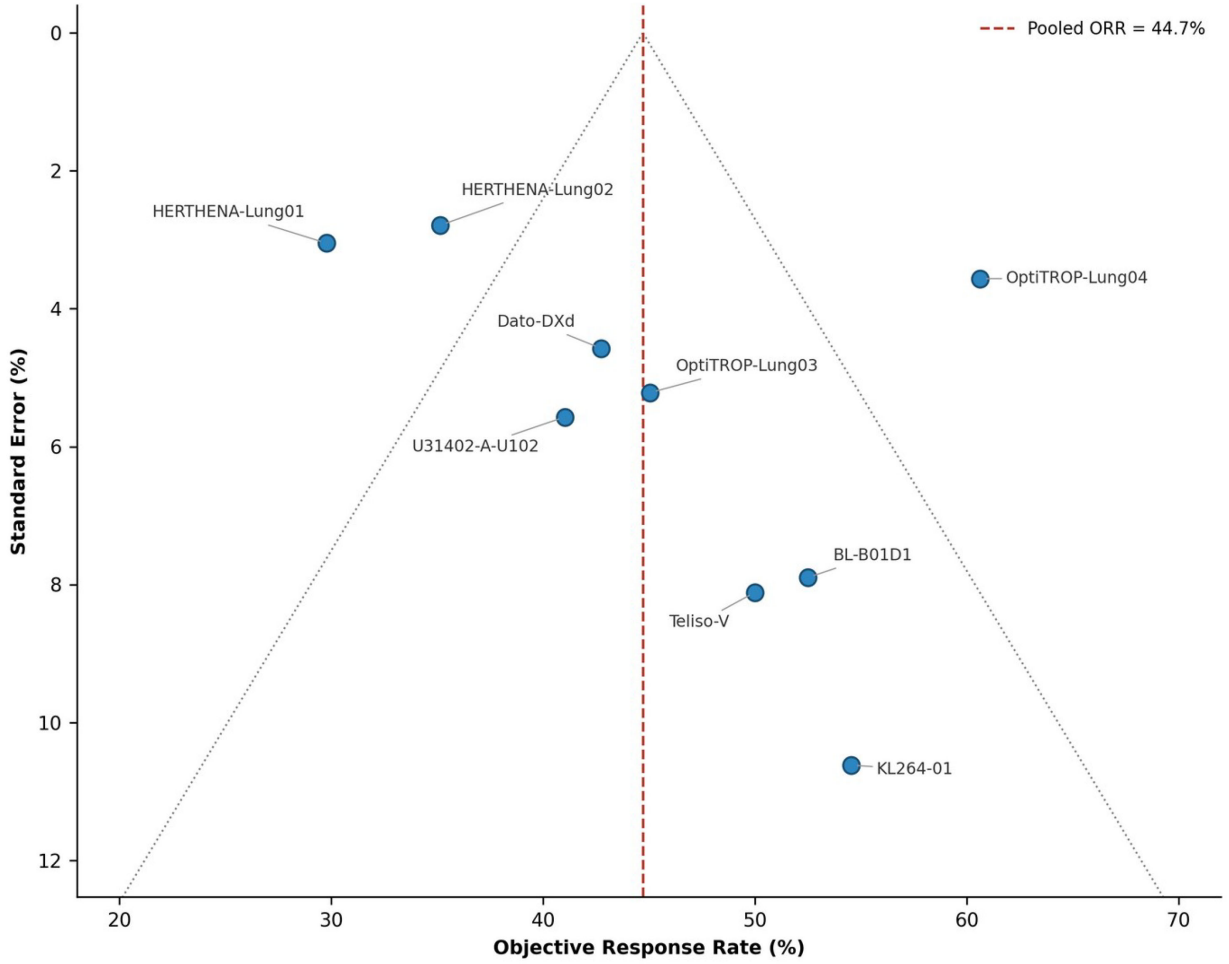
Funnel plot of the nine included studies for assessment of publication bias. The dashed line indicates the pooled ORR (44.7%); visual inspection suggests mild asymmetry.

**Figure 6:**
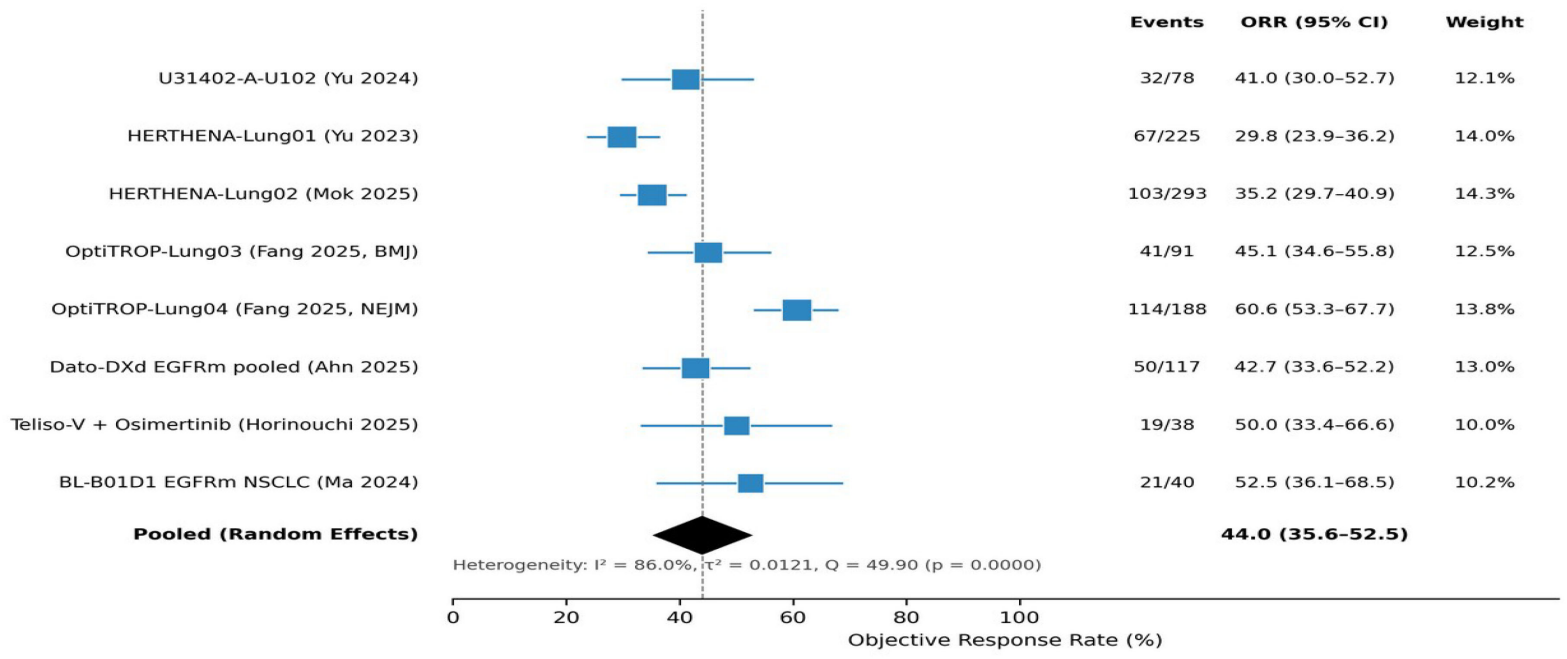
Sensitivity analysis forest plot after exclusion of KL264-01. The pooled ORR of the remaining eight studies was 44.0% (95% CI 35.6%–52.5%).

Leave-one-out sensitivity analysis demonstrated that the pooled ORR was robust, ranging from 41.2% to 47.0% upon sequential exclusion of each study. Exclusion of OptiTROP-Lung04 produced the largest downward shift (41.2%) and the greatest reduction in heterogeneity (I² = 63.7%), reflecting its large size and high ORR; exclusion of HERTHENA-Lung01 yielded 47.0% (I² = 78.9%). Exclusion of KL264-01, which had potential patient overlap with OptiTROP-Lung03, yielded a pooled ORR of 44.0%, consistent with the primary analysis. Visual inspection of the funnel plot (**Figure 5**) revealed mild asymmetry, although formal testing was not performed given the limited number of studies. A sensitivity forest plot excluding KL264–01 is shown in **Figure 6**.

### 3.8 Safety profile

Frequently reported adverse events included haematologic toxicities (neutropenia, thrombocytopenia, anaemia) and gastrointestinal events (nausea, stomatitis, diarrhoea). Interstitial lung disease (ILD)—a class-effect concern for DXd-payload ADCs—occurred at rates of 5–7% in HER3-DXd studies (adjudicated drug-related ILD in 5% of HER3-DXd patients in HERTHENA-Lung02, including grade 5 events) and at lower rates with TROP2-ADCs. In HERTHENA-Lung02, grade ≥3 treatment-emergent adverse events occurred in 73% of HER3-DXd patients versus 57% with chemotherapy, driven largely by grade ≥3 thrombocytopenia (30% vs 7.9%). Frequently reported ADC toxicities in this setting are predominantly haematologic and gastrointestinal (41). In OptiTROP-Lung04, the most common grade ≥3 treatment-related event was decreased neutrophil count (39.9%); TROP2-ADCs generally show a manageable, reversible haematologic profile (42). Owing to heterogeneous safety reporting, formal pooled safety analysis was not conducted; safety findings are descriptive (**Table 3**).

**Table 3:** Safety profile summary across included studies.

Study	Target	Common ≥G3 / notable AEs	ILD (any grade)	TRAE source
HERTHENA-Lung01	HER3	Neutropenia, thrombocytopenia, fatigue	5–7% (incl. 1 fatal)	21
U31402-A-U102	HER3	Thrombocytopenia, neutropenia	~5%	36
HERTHENA-Lung02	HER3	≥G3 TEAE 73% vs 57%; ≥G3 thrombocytopenia 30% vs 7.9%	5% (G1/2 11, G3 1, G5 2)	23
OptiTROP-Lung04	TROP2	≥G3 neutrophil count decreased 39.9%	Low	20 (NEJM)
OptiTROP-Lung03	TROP2	Reversible haematologic toxicity	Low	38 (BMJ)
BL-B01D1	EGFR×HER3	Neutropenia 47%, leukopenia 39% (overall)	NR	22

AE, adverse event; G, grade; ILD, interstitial lung disease; NR, not reported; TEAE, treatment-emergent adverse event; TRAE, treatment-related adverse event. The HERTHENA-Lung02 row (highlighted) is new in this revision. Owing to heterogeneous reporting, safety data are descriptive and were not pooled.

## 4 Discussion

This meta-analysis represents the first comprehensive evaluation of ADCs specifically in EGFR-mutant NSCLC following TKI resistance. The pooled ORR of 44.7% across 1,092 patients demonstrates that ADCs confer meaningful antitumour activity in this difficult-to-treat population, substantially exceeding the 10–20% response rates achieved with standard salvage chemotherapy (15, 16, 43).

First, the significant difference in ORR between TROP2- and HER3-targeting ADCs (50.6% vs 34.2%, P = 0.006) suggests that target selection carries meaningful clinical implications. This should be interpreted with caution, as three of the four TROP2 studies evaluated Sac-TMT, so the difference may partly reflect drug-specific properties. TROP2 is broadly overexpressed in NSCLC (~88% in advanced disease) (44), and preclinical evidence suggests EGFR mutations enhance internalisation and activity of TROP2-ADCs (37). In contrast, HER3 is widely expressed regardless of mutational status and is associated with metastasis and shorter recurrence-free survival (45); HER3-DXd shows activity across both known and unknown resistance mechanisms (46).

HER3 versus TROP2. A central observation from the updated evidence base is the divergence between HER3- and TROP2-targeting ADCs at the level of OS. Whereas sacituzumab tirumotecan achieved both PFS and OS benefit in OptiTROP-Lung04, patritumab deruxtecan in the larger phase III HERTHENA-Lung02 improved PFS (HR 0.77) but, at the protocol-specified interim analysis, OS data remained immature and no OS benefit had yet been demonstrated. Several non-mutually-exclusive factors may explain this discordance: extensive post-progression access to subsequent therapies in a predominantly Asian population may have diluted the OS signal; HER3 expression is largely independent of EGFR mutational status, whereas EGFR mutations may enhance TROP2-ADC internalisation and cytotoxic delivery; and differences in payload potency, linker chemistry, and delivered drug-to-antibody ratio may favour Sac-TMT. Together, these data argue against a class-level OS claim for ADCs and support a target- and drug-specific interpretation, with TROP2-directed agents currently providing the most robust randomized evidence in this population.

Drug-specific versus target-specific efficacy within TROP2. Within the TROP2 subgroup, Sac-TMT produced consistently higher ORRs (OptiTROP-Lung04 60.6%, OptiTROP-Lung03 45.1%, KL264–01 EGFRm 54.5%) than Dato-DXd (43%). Critically, only Sac-TMT was positive for OS, whereas TROPION-Lung01 (Dato-DXd, biomarker-unselected) did not meet its OS endpoint. This argues that the efficacy advantage is at least partly drug-specific—attributable to Sac-TMT’s distinct 2-methylsulfonylpyrimidine linker coupled to the belotecan-derivative payload KL610023 and its higher drug-to-antibody ratio—rather than purely target-specific. Quantitative TROP2 and HER3 expression have so far shown limited predictive value, and EGFR-mutation status itself may enrich for response; biomarker qualification remains an open need.

Second, the RCT data for Sac-TMT are particularly compelling. The pooled OS HR of 0.57 (I² = 0%) demonstrates a consistent survival benefit over chemotherapy, establishing Sac-TMT as the first TROP2-ADC to show OS improvement in EGFR-mutant NSCLC; OptiTROP-Lung04 has led to regulatory approval of Sac-TMT in China for this indication.

Third, emerging data on next-generation ADCs are encouraging. BL-B01D1, a first-in-class EGFR×HER3 bispecific ADC, achieved an ORR of 52.5%, with responses across diverse resistance mechanisms. Telisotuzumab vedotin plus osimertinib achieved an ORR of 50.0% in c-MET-overexpressing NSCLC after osimertinib failure, although the ADC contribution cannot be isolated; this supports targeting bypass pathways with ADCs, given that MET amplification is a well-established osimertinib-resistance mechanism (47). EGFR-targeting ADCs such as MRG003 (48) and SYS6010 (49) have shown preliminary activity (ORR 31–44%), and bispecific EGFR-cMET ADCs such as AZD9592 are in development (50).

### 4.1 Comparison with existing meta-analyses

Zhang et al. (24) reported a pooled ORR of 33% for ADC monotherapy in previously treated NSCLC, with higher ORRs in EGFR-mutant (35%) subgroups. Stumpo et al. (28) analysed TROP2-ADCs versus docetaxel and found a significant OS benefit in the actionable-genomic-alteration (AGA) subgroup but not the overall population. Khan et al. (51) analysed ADCs versus chemotherapy using three RCTs including EVOKE-01. Although EVOKE-01 (sacituzumab govitecan vs docetaxel; 52) did not meet its OS endpoint in the biomarker-unselected population, its EGFR/AGA subgroup—together with the AGA subgroup of TROPION-Lung01 (pooled OS HR 0.63)—showed a more favourable signal than the overall population, reinforcing the view that EGFR-mutant NSCLC is a preferentially ADC-sensitive subpopulation. The present meta-analysis is the first to focus exclusively on the EGFR-mutant population and to integrate data across multiple ADC targets.

### 4.2 Limitations

Several limitations warrant consideration. First, substantial heterogeneity (I² = 84%) reflects the diversity of ADC targets, treatment lines, and designs; we therefore emphasise target-stratified estimates over the global pooled value. Second, mature OS data were available from only three of nine studies, so broad survival inferences beyond the randomized Sac-TMT evidence should be made cautiously. Third, one ADC-plus-TKI combination study (telisotuzumab vedotin plus osimertinib) precludes isolation of the ADC effect; a sensitivity analysis excluding it left the pooled ORR essentially unchanged (44.2%). Fourth, most remaining studies were single-arm phase I–II trials susceptible to selection bias. Fifth, potential patient overlap may exist between the KL264–01 EGFRm subgroup and OptiTROP-Lung03; relatedly, the SKB264-II-08 cohort (n = 64, ORR 34%) reported in the same publication as KL264–01 was excluded to minimise correlated data. Sixth, the meta-regression of prior treatment lines was underpowered (k = 9). Seventh, publication bias could not be formally assessed given the limited number of studies.

### 4.3 Future directions

With HERTHENA-Lung02 now reported, the key remaining phase III questions concern Dato-DXd and combination strategies. TROPION-Lung14 and TROPION-Lung15 will evaluate Dato-DXd combined with osimertinib as first-line or post-osimertinib therapy, and OptiTROP-Lung05 is evaluating Sac-TMT plus pembrolizumab as first-line therapy in PD-L1-positive NSCLC. Bispecific ADCs (BL-B01D1, AZD9592) and novel EGFR-targeting ADCs represent another frontier. The HERTHENA-Lung02 experience—PFS benefit without OS benefit—highlights the importance of trial design choices (treatment line, crossover, endpoint selection) and of biomarker-driven selection for future HER3-ADC development. Biomarker-driven patient selection, optimal sequencing, and rational combinations will be critical areas of future investigation.

## 5 Conclusion

This systematic review and meta-analysis demonstrates that ADCs provide clinically meaningful antitumour activity in EGFR-mutant NSCLC following TKI resistance, with a pooled ORR of 44.7%. Efficacy is target- and drug-specific: only the TROP2-ADC sacituzumab tirumotecan has demonstrated improvement in both PFS and OS over chemotherapy, supported by phase III evidence, whereas the HER3-ADC patritumab deruxtecan improved PFS without an OS benefit. These findings establish ADCs—particularly TROP2-directed agents—as a key therapeutic pillar for EGFR-TKI-resistant NSCLC and provide a framework for biomarker-guided clinical decision-making and future trial design.

## References

[1] Thai AA Solomon BJ Sequist LV Gainor JF Heist RS . Lung cancer. Lancet. (2021) 398:535–54. doi: 10.1016/S0140-6736(21)00312-3 34273294

[2] Miller KD Nogueira L Devasia T Mariotto AB Yabroff KR Jemal A . Cancer treatment and survivorship statistic. CA Cancer J Clin. (2022) 72:409–36. doi: 10.3322/caac.21731 35736631

[3] Borgeaud M Olivier T Bar J Zer A Garinet S Loriot Y . Personalized care for patients with EGFR-mutant non-small cell lung cancer: navigating early to advanced disease management. CA Cancer J Clin. (2025) 75:387–409. doi: 10.3322/caac.21877 40673977 PMC12432819

[4] Pakkala S Ramalingam SS . Personalized therapy for lung cancer: striking a moving target. JCI Insight. (2018) 3:e120858. doi: 10.1172/jci.insight.120858 30089719 PMC6129126

[5] Soria JC Ohe Y Vansteenkiste J Reungwetwattana T Chewaskulyong B Lee KH . Osimertinib in untreated EGFR-mutated advanced non-small-cell lung cancer. N Engl J Med. (2018) 378:113–25. doi: 10.1056/NEJMoa1713137 29151359

[6] Duma N Santana-Davila R Molina JR . Non-small cell lung cancer: epidemiology, screening, diagnosis, and treatment. Mayo Clin Proc. (2019) 94:1623–40. doi: 10.1016/j.mayocp.2019.01.013 31378236

[7] Rotow J Bivona TG . Understanding and targeting resistance mechanisms in NSCLC. Nat Rev Cancer. (2017) 17:637–58. doi: 10.1038/nrc.2017.84 29068003

[8] Dong RF Zhu ML Liu MM Xu YT Yuan LL Bian J . EGFR mutation mediates resistance to EGFR tyrosine kinase inhibitors in NSCLC: from molecular mechanisms to clinical research. Pharmacol Res. (2021) 167:105583. doi: 10.1016/j.phrs.2021.105583 33775864

[9] Engelman JA Zejnullahu K Mitsudomi T Song Y Hyland C Park JO . MET amplification leads to gefitinib resistance in lung cancer by activating ERBB3 signaling. Science. (2007) 316:1039–43. doi: 10.1126/science.1141478 17463250

[10] Peters TL Patil T Le AT Pham TT Doebele RC . Evolution of MET and NRAS gene amplification as acquired resistance mechanisms in EGFR mutant NSCLC. NPJ Precis Oncol. (2021) 5:91. doi: 10.1038/s41698-021-00232-w 34642436 PMC8511249

[11] Ramalingam SS Cheng Y Zhou CC Ohe Y Imamura F Cho BC . Mechanisms of acquired resistance to first-line osimertinib: preliminary data from the phase III FLAURA study. Ann Oncol. (2018) 29:viii740. doi: 10.1093/annonc/mdy424.063

[12] Chmielecki J Mok T Wu YL Han JY Ahn MJ Ramalingam SS . Analysis of acquired resistance mechanisms to osimertinib in patients with EGFR-mutated advanced non-small cell lung cancer from the AURA3 trial. Nat Commun. (2023) 14:1071. doi: 10.1038/s41467-023-35962-x 36849516 PMC9971022

[13] Schoenfeld AJ Chan JM Kubota D Sato H Rizvi H Daneshbod Y . Tumor analyses reveal squamous transformation and off-target alterations as early resistance mechanisms to first-line osimertinib in EGFR-mutant lung cancer. Clin Cancer Res. (2020) 26:2654–63. doi: 10.1158/1078-0432.CCR-19-3563 31911548 PMC7448565

[14] Chmielecki J Gray JE Cheng Y Ohe Y Imamura F Cho BC . Candidate mechanisms of acquired resistance to first-line osimertinib in EGFR-mutated advanced non-small cell lung cancer. Nat Commun. (2023) 14:1070. doi: 10.1038/s41467-023-35961-y 36849494 PMC9971254

[15] Han B Yang L Wang X Yao L Jiang T . Efficacy of pemetrexed-based regimens in advanced non-small cell lung cancer patients with activating epidermal growth factor receptor mutations after tyrosine kinase inhibitor failure: a systematic review. OncoTargets Ther. (2018) 11:2121–9. doi: 10.2147/OTT.S154110 29695919 PMC5905532

[16] Hayashi H Nishio M Takahashi M Ozeki T Akamatsu H . Real-world data about treatment outcomes for patients with EGFR-mutated NSCLC resistance to osimertinib and platinum-based chemotherapy. Adv Ther. (2023) 40:4545–60. doi: 10.1007/s12325-023-02614-x 37572265 PMC10499725

[17] Fu Z Li S Han S Shi C Zhang Y . Antibody drug conjugate: the “biological missile” for targeted cancer therapy. Signal Transduc Tgt Ther. (2022) 7:93. doi: 10.1038/s41392-022-00947-7 35318309 PMC8941077

[18] Hsu R Benjamin DJ . A narrative review of antibody-drug conjugates in EGFR-mutated non-small cell lung cancer. Front Oncol. (2023) 13:1252652. doi: 10.3389/fonc.2023.1252652 38107063 PMC10722249

[19] Deng S Sun C Liu D Li J Xu G . Antibody-drug conjugates: a new twist to overcome EGFR-TKIs resistance in non-small cell lung cancer. Pharmacol Res. (2026) 223:108066. doi: 10.1016/j.phrs.2025.108066 41391577

[20] Fang W Yang Y Luo Y Zhang L Wu L Zhou C . Sacituzumab tirumotecan in EGFR-TKI-resistant NSCLC. N Engl J Med. (2025) 394(1):13–26. doi: 10.1056/NEJMoa2512071 41124220

[21] Yu HA Goto Y Hayashi H Felip E Cheng Y Thongprasert S . HERTHENA-Lung01, a phase II trial of patritumab deruxtecan (HER3-DXd) in epidermal growth factor receptor-mutated non-small-cell lung cancer after epidermal growth factor receptor tyrosine kinase inhibitor therapy and platinum-based chemotherapy. J Clin Oncol. (2023) 41:5363–75. doi: 10.1200/JCO.23.01476 37689979 PMC10713116

[22] Ma Y Huang Y Zhao Y Liu S Ding J Zhang Z . BL-B01D1, a first-in-class EGFR-HER3 bispecific antibody-drug conjugate, in patients with locally advanced or metastatic solid tumours: a first-in-human, open-label, multicentre, phase 1 study. Lancet Oncol. (2024) 25:901–11. doi: 10.1016/S1470-2045(24)00124-7 38823410

[23] Mok TSK Yu HA Lim SM Okamoto I Perol M Novello S . Patritumab deruxtecan (HER3-DXd) in resistant EGFR-mutated (EGFRm) advanced non-small cell lung cancer (NSCLC) after a third-generation EGFR TKI: the phase 3 HERTHENA-Lung02 study. J Clin Oncol. (2025) 43:8506. doi: 10.1200/JCO.2025.43.16_suppl.8506 42148471

[24] Zhang L Jia H Niu B . Evaluating the efficacy and safety of antibody-drug conjugates in non-small cell lung cancer: a systematic review and meta-analysis. BMC Cancer. (2026) 26:228. doi: 10.1186/s12885-025-15461-6 41545863 PMC12896352

[25] Jin X Zhao W Li B Gu Y Li Z Guo W . Efficacy and safety of antibody-drug conjugates in the treatment of non-small cell lung cancer: a systematic review and meta-analysis of prospective clinical trials. Transl Cancer Res. (2025) 14:5255–70. doi: 10.21037/tcr-2025-376 41158249 PMC12554454

[26] Zhao L Wang J Chen H . Antibody drug conjugates demonstrate potential as HER-2-targeted therapies for NSCLC. Oncol Lett. (2025) 30:481. doi: 10.3892/ol.2025.14727 40861101

[27] Li Y Zhou L Li S Zhou S Chen Y Tong Y . Treatment-related adverse events of antibody-drug conjugate monotherapy in non-small cell lung cancer: a systematic review and meta-analysis. Thorac Cancer. (2025) 16:e70178. doi: 10.1111/1759-7714.70178 41268683 PMC12635590

[28] Stumpo S Carlini A Mantuano F Di Federico A Melotti B Sperandi F . Efficacy and safety of TROP-2-targeting antibody-drug conjugate treatment in previously treated patients with advanced non-small cell lung cancer: a systematic review and pooled analysis of reconstructed patient data. Cancers. (2025) 17:1750. doi: 10.3390/cancers17111750 40507234 PMC12153610

[29] Page MJ McKenzie JE Bossuyt PM Boutron I Hoffmann TC Mulrow CD . The PRISMA 2020 statement: an updated guideline for reporting systematic reviews. BMJ. (2021) 372:n71. doi: 10.1136/bmj.n71 33782057 PMC8005924

[30] Sterne JAC Savović J Page MJ Elbers RG Blencowe NS Boutron I . RoB 2: a revised tool for assessing risk of bias in randomised trials. BMJ. (2019) 366:l4898. doi: 10.1136/bmj.l4898 31462531

[31] Wells GA Shea B O’Connell D Peterson J Welch V Losos M . The Newcastle-Ottawa Scale (Nos) for Assessing the Quality of Nonrandomised Studies in Meta-Analyses. Ottawa: Ottawa Hospital Research Institute (2014).

[32] Freeman MF Tukey JW . Transformations related to the angular and the square root. Ann Math Stat. (1950) 21:607–11. doi: 10.1214/aoms/1177729756

[33] DerSimonian R Laird N . Meta-analysis in clinical trials. Ctrl Clin Trials. (1986) 7:177–88. doi: 10.1016/0197-2456(86)90046-2 3802833

[34] Higgins JPT Thomas J Chandler J Cumpston M Li T Page MJ . Cochrane Handbook for Systematic Reviews of Interventions, Version 6.5. London: Cochrane (2024).

[35] Higgins JP Thompson SG . Quantifying heterogeneity in a meta-analysis. Stat Med. (2002) 21:1539–58. doi: 10.1002/sim.1186 12111919

[36] Yu HA Baik C Kim DW Johnson ML Hayashi H Nishio M . Translational insights and overall survival in the U31402-A-U102 study of patritumab deruxtecan (HER3-DXd) in EGFR-mutated NSCLC. Ann Oncol. (2024) 35:437–47. doi: 10.1016/j.annonc.2024.01.010 38369013

[37] Zhao S Cheng Y Wang Q Zhang L Fang W Wu L . Sacituzumab tirumotecan in advanced non-small-cell lung cancer with or without EGFR mutations: phase 1/2 and phase 2 trials. Nat Med. (2025) 31(6):1976–86. doi: 10.1038/s41591-025-03638-2 40210967

[38] Fang W Li X Wang Q Zhang Y Ma Y Zhong W . Sacituzumab tirumotecan versus docetaxel for previously treated EGFR-mutated advanced non-small cell lung cancer: multicentre, open label, randomised controlled trial. BMJ. (2025) 389:e085680. doi: 10.1136/bmj-2025-085680 40473437 PMC12139608

[39] Ahn MJ Lisberg A Goto Y Paz-Ares L Camidge DR Sands J . A pooled analysis of datopotamab deruxtecan in patients with EGFR-mutated NSCLC. J Thorac Oncol. (2025) 20(11):1669–82. doi: 10.1016/j.jtho.2025.06.002 40516821

[40] Horinouchi H Cho BC Camidge DR Kato T Reckamp KL Yang JC . Results from a phase 1b study of telisotuzumab vedotin in combination with osimertinib in patients with c-Met protein-overexpressing, EGFR-mutated locally advanced/metastatic non-small cell lung cancer after progression on prior osimertinib. Ann Oncol. (2025) 36(5):583–91. doi: 10.1016/j.annonc.2025.01.001 39805351

[41] Passaro A Jänne PA Peters S . Antibody-drug conjugates in lung cancer: recent advances and implementing strategies. J Clin Oncol. (2023) 41:3747–61. doi: 10.1200/JCO.23.00013 37224424

[42] Li M Jin M Peng H Zhang Y Wang C . Current status and future prospects of TROP-2 ADCs in lung cancer treatment. Drug Des Devel Ther. (2024) 18:5005–21. doi: 10.2147/DDDT.S492200 39525044 PMC11550919

[43] Johnson M Garassino MC Mok T Mitsudomi T . Treatment strategies and outcomes for patients with EGFR-mutant non-small cell lung cancer resistant to EGFR tyrosine kinase inhibitors: focus on novel therapies. Lung Cancer. (2022) 170:41–51. doi: 10.1016/j.lungcan.2022.05.011 35714425

[44] Omori S Muramatsu K Kawata T Akamatsu H Toi Y . Trophoblast cell-surface antigen 2 expression in lung cancer patients and the effects of anti-cancer treatments. J Cancer Res Clin Oncol. (2022) 148:2455–63. doi: 10.1007/s00432-022-04093-z 34533624 PMC11801068

[45] Li Q Zhang R Yan H Zhao P Wu L Wang H . Prognostic significance of HER3 in patients with Malignant solid tumors. Oncotarget. (2017) 8:67140–51. doi: 10.18632/oncotarget.18007 28978022 PMC5620162

[46] Jänne PA Baik C Su WC Johnson ML Hayashi H Nishio M . Efficacy and safety of patritumab deruxtecan (HER3-DXd) in EGFR inhibitor-resistant, EGFR-mutated non-small cell lung cancer. Cancer Discov. (2022) 12:74–89. doi: 10.1158/2159-8290.CD-21-0715 34548309 PMC9401524

[47] Leonetti A Sharma S Minari R Perego P Giovannetti E Tiseo M . Resistance mechanisms to osimertinib in EGFR-mutated non-small cell lung cancer. Br J Cancer. (2019) 121:725–37. doi: 10.1038/s41416-019-0573-8 31564718 PMC6889286

[48] Qiu MZ Zhang Y Guo Y Guo W Nian W Liao M . Evaluation of safety of treatment with anti-epidermal growth factor receptor antibody drug conjugate MRG003 in patients with advanced solid tumors: a phase 1 nonrandomized clinical trial. JAMA Oncol. (2022) 8:1042–6. doi: 10.1001/jamaoncol.2022.1238 35511148 PMC9073657

[49] Lu S Zhou Z Li ZM Chen J Wu YL . First-in-human study of SYS6010, a novel EGFR targeting antibody drug conjugate (ADC) for patients with advanced solid tumors. Cancer Res. (2025) 85:CT008. doi: 10.1158/1538-7445.AM2025-CT008 36230740

[50] Comer F Mazor Y Hurt E Yang Y Bhatt D Bhagwat S . AZD9592: an EGFR-cMET bispecific antibody-drug conjugate (ADC) targeting key oncogenic drivers in non-small-cell lung cancer (NSCLC) and beyond. Cancer Res. (2023) 83:5736. doi: 10.1158/1538-7445.AM2023-5736 36230740

[51] Khan SR Eiras LM Boldt G Raphael J Breadner D . Antibody-drug conjugates versus chemotherapy in previously treated non-small-cell lung cancer: a systematic review and meta-analysis. Ther Adv Med Oncol. (2025) 17:1–18. doi: 10.1177/17588359251387393 41179119 PMC12579195

[52] Paz-Ares LG Juan-Vidal O Mountzios GS Kato T Prabhash K Syrigos K . Sacituzumab govitecan versus docetaxel for previously treated advanced or metastatic non-small cell lung cancer: the randomized, open-label phase III EVOKE-01 study. J Clin Oncol. (2024) 42:2860–72. doi: 10.1200/JCO.24.00733 38843511 PMC11328920

